# Carotid intima medial thickness and its association with cardiometabolic risk factors in children with overweight and obesity: a hospital-based cross-sectional study

**DOI:** 10.1017/S0007114525000091

**Published:** 2025-02-28

**Authors:** Sabitha Sasidharan Pillai, M. Vijayakumar, Ajitha Balakrishnan

**Affiliations:** 1 Department of Pediatrics, Government Medical College, Kozhikode, Kerala, India; 2 Center for Endocrinology, Diabetes and Metabolism, Children’s Hospital Los Angeles, Los Angeles, CA, Department of Pediatrics, Keck School of Medicine, University of Southern California, Los Angeles, CA, USA; 3 Department of Community Medicine, Government Medical College, Thrissur, Kerala, India

**Keywords:** Children, Adolescents, Obesity, Overweight, Carotid intima media thickness

## Abstract

A hospital-based cross-sectional study involving children aged 2–15 years attending the obesity clinic of a tertiary care hospital from January 2016 to March 2018 was carried out to study carotid intima media thickness (cIMT) and its association with cardiometabolic risk factors in children with overweight and obesity. Secondary objective was to compare children with elevated (EcIMT) and normal cIMT (NcIMT). Out of 223 patients enrolled for the study, 102 (45·7 %) had EcIMT. Mean cIMT of the study participants was 0·41 (sd 0·13) mm. Median alanine transaminase levels (27 *v*. 24, *P*= 0·006) and proportion of patients with fatty liver (63·7 % *v*. 48·8 %, *P*= 0·025) and ≥ 3 risk factors (80·4 % *v*. 66·1 %, *P*= 0·003) were higher in the EcIMT group compared with NcIMT group. Proportion of patients with hypercholesterolemia (36·4 % *v*. 16 %, *P*= 0·024), elevated LDL-cholesterol (38·6 % *v*. 16 %, *P*= 0·013), low HDL-cholesterol (40·9 % *v*. 20 %, *P*= 0·027) and dyslipidemia (84·1 % *v*. 58 %, *P*= 0·006) was higher in the pubertal EcIMT group and those with fatty liver (63·8 % *v*. 45·1 %, *P*= 0·034) was higher in the prepubertal EcIMT group compared with pubertal and prepubertal NcIMT groups, respectively. No significant correlations were observed between cIMT and various cardiometabolic parameters. Our finding of EcIMT in nearly half of the study participants including young children is very concerning as these children are at increased risk of atherosclerotic CVD in adulthood. Interventions starting at a young age are important when trajectories are likely to be more malleable and adverse cardiometabolic phenotypes and subclinical atherosclerosis are reversible.

The prevalence of obesity among paediatric population has increased by more than 8-fold over the last 4 decades and is a significant public health problem globally^(1)^. Children with obesity are at increased risk for cardiovascular morbidity and mortality in early adulthood. Carotid intima media thickness (cIMT) is considered as an early marker of carotid arterial injury and subclinical atherosclerosis that precedes plaque formation. Subclinical atherosclerosis may start at a young age in conditions at increased risk for atherosclerotic CVD such as obesity^(2)^. Chronic inflammation contributes to the progression of atherosclerosis, and risk factors enhance the pathogenesis by hastening the underlying inflammatory process^(3)^. Non-invasive assessment of cIMT with high-resolution B-mode ultrasonography helps to identify early atherosclerosis^(3)^. Prior studies observed that children with obesity and increased cIMT were at higher risk for atherosclerotic CVD in adulthood compared with children without obesity^(4)^. Early recognition of risk factors of vascular health in children is important as the initial lesions of atherosclerosis can be revocable with lifestyle modification^(5)^. There have been few studies on cIMT in children with obesity from South Asia and none in children younger than 5 years of age from Asia^(6–9)^. We aimed to study cIMT and its association with cardiometabolic risk factors in children with obesity and to compare children with elevated (EcIMT) and normal cIMT (NcIMT). We hypothesised that among children with obesity metabolic abnormalities will be higher in those with EcIMT compared with those with NcIMT.

## Methods

This hospital-based cross-sectional study was conducted in the Department of Pediatrics, Government Medical College, Kozhikode, Kerala, India. Children whose parents had given written consent were included in the study as per the following criteria: children aged 2–15 years with overweight or obesity due to exogenous causes attending the paediatric obesity clinic during the period from 1 January 2016 to 31 March 2018. Children < 13 years of age are admitted in the paediatric words of government hospitals in Kerala, India, as per the government policy, and those ≥ 13 years are admitted and managed in the adult wards. Though both paediatric and adult outpatient specialty clinics could see patients between 13 years and 21 years of age,most patients >15 years of age preferred adult specialty clinics. Hence, patients > 15 years were not included in our study. Based on a previous study^(6)^, the mean cIMT of children with overweight and obesity was 0·5 (sd 0·1) cm. So, we calculated that for similar results with a marginal error 0·02 mm in mean cIMT, a sample size of 196 would be sufficient for 5 % significance level and 80 % power. Overweight and obesity were defined as per the Indian Academy of Pediatrics (IAP) 2015 BMI charts/age for boys and girls, respectively (BMI reference curves of adult equivalent cut-off 23 and 27 were considered as overweight and obesity, respectively) for children aged 5–15 years. The 23 and 27 adult equivalent cut-offs lines are suggested by the IAP to define overweight and obesity, respectively, as Asians are known to have more adiposity and increased cardiometabolic risk at a lower BMI. For children < 5 years of age, overweight and obesity were defined as per the WHO growth charts for boys and girls, respectively: overweight with BMI-for-age > 1 sd above the WHO Growth Reference median; obesity with BMI > 2 sd above the WHO Growth Reference median^(10,11)^. The exclusion criteria were children with genetic, endocrine and pharmacological causes of overweight/obesity or recent history of acute infectious or non-infectious inflammatory disorders. Preformed questionnaire was used to collect clinical and demographic data. General and systemic examination were performed in all the patients. Electronic weighing machine (Easy Care) was used to measure body weight to an accuracy of 0·1 kg with the participants wearing light clothes and no foot wears. The height was taken using a stadiometer (Prestige height measuring scale, C-117, Mayapuri Industrial Area; range, 20–210 cm) to an accuracy of 0·1 cm. Waist circumference (WC) was measured midway between the lowest rib cage and the iliac crest with a non-stretchable tape, to the nearest 0·1 cm, with the subject in a standing position and no clothes covering the measuring area. Pan Indian WC data that represented age- and sex-specific WC percentiles for Indian children aged 2–18 years were used for WC and the WC percentile > 70 percentile for age and sex was taken as the cut-off^(12)^. Pubertal assessment was done using Tanner’s sexual maturity rating: Tanner stage 1 being prepubertal and Tanner stage 2–5 pubertal^(13,14)^. Blood pressure was measured by a trained investigator on the right upper arm with the study participant in the sitting position. Measurements were made by auscultation with a mercury-column sphygmomanometer (ELKOMETER, Anita Industries). The average of three consecutive measurements was used for analysis. Blood pressure ≥ 90th percentile and > 95th percentile for age, sex and height were taken as elevated blood pressure and hypertension, respectively^(15)^.

Blood was collected after an overnight fast for fasting blood glucose (FBG) plasma insulin, lipid profile, alanine transaminase (ALT), aspartate transaminase (AST), uric acid and HbA1c. FBG was measured by glucose oxidase-peroxidase method in HITACHI 912 autoanalyzer using colorimetric method. Fasting insulin was measured with Roche Elecsys 2010 electrochemiluminescent autoanalyzer with an immunoassay format. AST, ALT, uric acid, total cholesterol (TC), triacylglycerol (TAG), HDL-cholesterol and VLDL-cholesterol were measured with an automated analyser and LDL-cholesterol was calculated by Friedewald equation^(16)^. HbA1c was measured by the Tosoh Automated Glycohemoglobin Analyzer HLC-723G8 using ion-exchange affinity chromatography method.

A diagnosis of metabolic syndrome (MS) was made in children aged 10 years and above in the presence of central adiposity^(12)^ and any two of the following criteria: (1) TAG levels > 95th percentile (0–9 years ≥ 100 mg/dl; 10–19 years ≥ 130 mg/dl), (2) HDL-cholesterol level < 40 mg/dl)^(17)^, (3) systolic or diastolic blood pressure ≥ 90th percentile for the age, sex and height^(15)^ and (4) FBG ≥ 100 mg/dl (impaired fasting glucose)^(18)^.

Hypercholesterolemia (TC ≥ 200 mg/dl), hypertriacylglycerolaemia, high LDL-cholesterol (≥ 130 mg/dl) or low HDL-cholesterol were accepted as dyslipidemia^(17)^. HbA1c < 5·7 % was taken as normal^(19)^. Hyperuricemia was diagnosed based on the Mayo clinic laboratories reference value for boys and girls of different age groups^(20)^. The degree of insulin resistance (IR) was determined by the homeostatic model assessment-IR (HOMA-IR), and HOMA-IR ≥ 2·5 is taken as abnormal^(21)^. Fasting insulin level > 15 mIU/ml in prepubertal children and > 26 mIU/ml in pubertal children was considered as hyperinsulinemia^(22)^. Caliper database that provides a summary of age- and sex-partitioned paediatric reference intervals for ALT was used to analyse ALT values^(23)^. Metabolic dysfunction associated fatty liver disease was diagnosed based on abnormal ultrasound or ALT greater than twice the upper limit of normal for the age and sex (> 50 IU/L for children < 13 years, 44 IU/L for females 13–18 years and 48 IU/L for males aged 13–16 years)^(23)^.

A longitudinal view of the distal common carotid artery was obtained from the suprasternal notch with a duplex scanner using a 7·5 MHz sector transducer in supine position. The cIMT measurements were made in both carotid arteries at 2 cm before bifurcation, and average was used for analysis. All B-mode carotid measurements were done by an experienced radiologist. cIMT measurements were analysed using the percentile curves proposed by Doyon *et al* ^(24)^. EcIMT is defined as cIMT > 75th percentile for age and sex in children aged 6–15 years^(25)^. Cut-off value for normal cIMT *v*. increased cIMT in children below 6 years was taken as the 75th percentile cIMT value for 6 years (0·36 mm for girls and 0·4 mm for boys). Study subjects were grouped into NcIMT group and EcIMT group based on the cIMT values. Patients were also grouped based on their pubertal status into prepubertal and pubertal groups. Each group was assessed for the prevalence of cardiometabolic risk factors in those with EcIMT compared with those with NcIMT and the correlations between the cardiometabolic parameters and cIMT. Based on cIMT, the study population was divided into quartiles and compared with clinical and cardiometabolic factors. The study was reviewed and approved by the institutional ethical board, IEC number GMCKKD/RP 2018/IEC/191.

### Statistical analysis

Statistical analysis was done using SPSS version 18.0 for Windows. Categorical data were expressed as number (frequency) and percentage. Quantitative data were expressed as mean (sd) if data are symmetric and as median with interquartile range if data are not symmetric. Categorical data were compared using *χ* ^2^ test. Quantitative data were compared using independent *t* test if data satisfied normality and using Mann–Whitney test if data did not satisfy normality. Based on cIMT, the patients were divided into quartiles: < = 0·3, 0·31–0·4, 0·41–0·5 and > 0·5. ANOVA or Kruskal–Wallis test was used to compare between these groups for normal or non-normal data, respectively. Pearson’s correlation was used to assess the correlation between normal variables, and Spearman’s rank correlation was used to assess the correlation between non-normal variables. Linear regression analysis was done to determine the predictors of cIMT. Logistic regression was used to determine the risk factors for EcIMT. Two-way ANOVA test was performed to assess the effect of pubertal stage and cIMT on various cardiometabolic parameters. All tests were two-sided, and a *P* value < 0·05 was considered as statistically significant.

## Results

Among the children attending the obesity clinic of a tertiary care hospital during the study period of 2 ¼ years, 223 children aged 2–15 years were enrolled for the study. Majority of the study population were between 6 and 10 years of age (*n* 109, 48·9 %), males (*n* 139, 62·7 %) and prepubertal (*n* 129, 57·8 %) and had obesity (*n* 180, 80·7 %). Mean cIMT of the study participants was 0·41 (sd 0·13) mm. EcIMT was seen in 102 children (45·7 %).

EcIMT group was compared with NcIMT group for clinical and metabolic parameters (Table 1) and metabolic abnormalities (Table 2). Median ALT levels were significantly higher in the EcIMT group compared with NcIMT group (27 *v*. 24, *P*= 0·006). Proportion of patients with fatty liver (63·7 % *v*. 48·8 %, *P*= 0·025) and ≥ 3 risk factors (80·4 % *v*. 66·1 %, *P*= 0·003) was significantly higher in the EcIMT group compared with NcIMT group. No statistically significant correlations were observed between cIMT and various clinical and cardiometabolic parameters (Table 3). No predictors for cIMT were identified on linear regression analysis. Sex or Tanner staging had no influence on cIMT. TC, TAG and LDL-cholesterol levels were higher in the EcIMT group compared with NcIMT group, but not statistically significant. Proportion of patients with hypertension, elevated systolic blood pressure (SBP), abnormal HbA1c, hyperinsulinemia, elevated ALT levels, hypercholesterolemia, hypertriacylglycerolaemia, elevated LDL-cholesterol, low HDL-cholesterol and dyslipidemia was higher in the EcIMT group compared with NcIMT group though not statistically significant. Out of 132 children aged ≥ 10 years, thirty had MS. Among these thirty children, sixteen had EcIMT (53·3 %) compared with forty-one children out of 102 who did not have MS (40·2 %) (*P*= 0·287).

**Table 1.** Clinical and demographic data of study participants (Numbers and percentages; median values and interquartile ranges; mean values and standard deviations)

	Total (*n* 223)		Participants with normal cIMT (*n* 121)		Participants with elevated cIMT (*n* 102)			
Patient characteristics	Median	IQR	Median	IQR	Median	IQR	*P*	
Age (years)	10·5	3·8	10·6	4·3	10·4	3·5	0·651	0·107
	*n*	%	*n*	%	*n*	%		
2–5 years (*n* (%))	23	10·3 %	13	10·7 %	10	9·8 %	0·924	
6–10 years	109	48·9 %	60	49·6 %	49	48 %		
11–15 years	91	40·8 %	48	39·7 %	43	42·2 %		
Female (*n* (%))	84	37·3 %	44	36·4 %	40	39·2 %	0·661	0·95
Male	139	62·7 %	77	63·6 %	62	60·8 %		
Patients with obesity (*n* (%))	180	80·7 %	98	81 %	82	80·4 %	0·91	
Overweight	43	19·3 %	23	19 %	20	19·6 %		
BMI (kg/m^2^)								
Mean	24·1		24·0		24·3		0·431	0·997
sd	3·3		3·3		3·3			
Birth weight (kg)								
Mean	3·0		3·0		2·95		0·515	
sd	0·77		0·68		0·55			
FH of hypertension (*n* (%))	141	63·2 %	82	67·8 %	59	57·8 %	0·126	
FH of dyslipidemia (*n* (%))	118	52·9 %	67	55·4 %	51	50 %	0·423	
FH of T2D (*n* (%))	160	71·7 %	84	69·4 %	76	74·5 %	0·4	
FH of heart attack (*n* (%))	87	39 %	50	41·3 %	37	36·3 %	0·441	
WC (cm)								
Mean	84		83·8		84·2		0·789	
sd	12		9·5		9·5			
	Median	IQR	Median	IQR	Median	IQR		
SBP (mm Hg)	100	20	100	20	100	16	0·193	
DBP (mm Hg)	62	10	62	10	62	10	0·889	
Prepubertal (*n*)								
*n*	129		71		58		0·785	
%	57·8 %		58·7 %		56·9 %			
Pubertal (*n*)								
*n*	94		50		44			
%	42·2 %		41·3 %		43·1 %			
AST (IU/L)	26	12	25	11	26	14·1	0·294	
ALT (IU/L)	25·6	21	24	16	27	26	0·006	0·134
Uric acid (mg/dl)	4·3	1·3	4·3	1·2	4·3	1·5	0·814	
FBG (mg/dl)								
Mean	86·1		86·5		85·4		0·397	
sd	13·0		11·3		8·9			
Fasting insulin (mIU/L)	13·4	10·6	13·3	9·1	13·6	11·4	0·544	
HOMA-IR	2·8	2·3	2·77	2·03	2·79	2·65	0·835	
HbA1c %	5·4	0·6	5·3	0·6	5·4	0·65	0·75	
TC (mg/dl)								
Mean	178		175·9		180·6		0·297	
sd	33·1		32·9		33·3			
TAG (mg/dl)	106·9	67	104	62	112	79·5	0·242	
LDL-cholesterol (mg/dl)								
Mean	111·9		110·2		114·1		0·328	
sd	29·8		30·7		28·8			
VLDL-cholesterol (mg/dl)	21	12·8	20	12	22	15	0·18	
HDL-cholesterol (mg/dl)								
Mean	42·6		42·9		42·3		0·567	
sd	8·3		7·8		8·9			

cIMT, carotid intima media thickness; FH, family history; T2D, type 2 diabetes; WC, waist circumference; SBP, systolic blood pressure; DBP, diastolic blood pressure; AST, aspartate transaminase; ALT, alanine transaminase; FBG, fasting blood glucose; HOMA-IR, homeostatic model assessment of insulin resistance; TC, total cholesterol; VLDL-cholesterol, very low-density lipoprotein cholesterol.

All values are expressed as median (IQR) unless otherwise specified. Adjusted *P* value is given in bracket.

**Table 2.** Cardiometabolic abnormalities in patients with elevated cIMT *v*. patients with normal cIMT (Numbers and percentages)

	Total patients (*n* 223)		Patients with normal cIMT (*n* 121)		Patients with elevated cIMT (*n* 102)			
Cardiometabolic abnormalities	*n*	%	*n*	%	*n*	%	P	
Central adiposity (*n* (%))	213	95·5 %	117	96·7 %	96	94·1 %	0·518	
Hypertension (*n* (%))	35	15·7 %	18	14·9 %	17	16·7 %	0·714	
Elevated SBP (*n* (%))	17	7·6 %	7	5·8 %	10	9·8 %	0·259	
Elevated DBP (*n* (%))	27	12·1 %	14	11·6 %	13	12·7 %	0·789	
IFG (*n* (%))	21	9·4 %	13	10·7 %	8	7·8 %	0·46	
HbA1c ≥ 5·7 % (*n* (%))	64	28·7 %	33	27·3 %	31	30·4 %	0·608	
HOMA-IR ≥ 2·5 (*n* (%))	131	58·7 %	75	62 %	56	54·9 %	0·285	
Hyperinsulinism (*n* (%)	54	24·2 %	25	20·7 %	29	28·4 %	0·177	
Elevated ALT (*n* (%))	111	49·8 %	56	46·3 %	55	53·9 %	0·256	
Hyperuricemia (*n* (%))	6	2·7 %	3	2·5 %	3	2·9 %	1·0	
Hypercholesterolemia (*n* (%))	57	25·6 %	27	22·3 %	30	29·4 %	0·226	
Hypertriacylglycerolaemia (*n* (%))	100	44·8 %	50	41·3 %	50	49·0 %	0·25	
Elevated LDL-cholesterol (*n* (%))	57	25·6 %	29	23·9 %	28	27·5 %	0·552	
Low HDL-cholesterol (*n* (%))	73	32·7 %	34	28·1 %	39	38·6 %	0·108	
Dyslipidemia (*n* (%))	151	67·7 %	79	65·3 %	72	70·6 %	0·399	
Fatty liver (*n* (%))	124	55·6 %	59	48·8 %	65	63·7 %	0·025*	0·18
1 risk factor present	20	9 %	9	7·4 %	11	10·8 %	0·003	0·485
2 risk factors present	42	18·4 %	32	26·4 %	9	8·8 %		
>= 3 risk factors present	162	72·6 %	80	66·1 %	82	80·4 %		

cIMT, carotid intima media thickness; SBP, systolic blood pressure; DBP, diastolic blood pressure; IFG, impaired fasting glucose; HOMA-IR, homeostatic model assessment of insulin resistance; ALT, alanine transaminase.

*Statistically significant at 5 % level. Adjusted *P* value is given in bracket.

**Table 3.** Correlation between cIMT and clinical and cardiometabolic parameters

Clinical and cardiometabolic parameter	Correlation coefficient (r)	*P*
Age	0·12^*^	0·08
BMI	0·06	0·40
Birth weight	0·02	0·75
WC	0·05	0·44
SBP	0·12^*^	0·09
DBP	0·03^*^	0·80
AST	–0·07^*^	0·82
ALT	0·13^*^	0·05
Uric acid	0·02^*^	0·79
FBG	–0·04	0·53
HOMA-IR	0·06^*^	0·34
Fasting insulin	0·06^*^	0·36
HbA1c	0·07^*^	0·33
TC	0·02	0·82
TAG	0·09^*^	0·17
LDL-cholesterol	–0·01	0·89
VLDL-cholesterol	0·08^*^	0·22
HDL-cholesterol	0·00	1·00

cIMT, carotid intima media thickness; WC, waist circumference; SBP, systolic blood pressure; DBP, diastolic blood pressure; AST, aspartate transaminase; ALT, alanine transaminase; FBG, fasting blood glucose; HOMA-IR, homeostatic model assessment of insulin resistance; TC, total cholesterol; VLDL-cholesterol, very low-density lipoprotein cholesterol.

* Spearman’s correlation coefficient, others Pearson’s correlation coefficient.

The prevalence of cardiometabolic risk factors was assessed separately in prepubertal and pubertal patients with normal and EcIMT (online Supplementary Table 1). Among pubertal group (*n* 94), proportion of patients with hypercholesterolemia (36·4 % *v*. 16 %, *P*= 0·024), elevated LDL-cholesterol (38·6 % *v*. 16 %, *P*= 0·013), low HDL-cholesterol (40·9 % *v*. 20 %, *P*= 0·027) and dyslipidemia (84·1 % *v*. 58 %, *P*= 0·006) were significantly higher in the EcIMT group compared with NcIMT group. Binary logistic regression with these variables was performed, but the model was not statistically significant, R^2^ = 0·127, *P*= 0·975. Among prepubertal group, proportion of patients with fatty liver was significantly higher in the EcIMT group compared with NcIMT group (63·8 % *v*. 45·1 %, *P*= 0·034). No statistically significant correlations were noted between cIMT and clinical and cardiometabolic parameters in both the prepubertal and pubertal groups (online Supplementary Table 2).

A separate analysis was done with children below 6 years of age to assess the correlation between cIMT and clinical and cardiometabolic parameters. In this age group, only age was found to be significantly correlated with cIMT (online Supplementary Table 3). A linear regression model was developed with age as predictor, and the model was statistically significant, R^2^ = 0·206, *P*= 0·03. The regression equation was cIMT = 0·212 + 0·041 × age. The projected cIMT level of children below age 6 years based on this model was 0·46 mm at age 6 years which was higher than the 75th percentile cIMT value for 6 years (0·36 mm for girls and 0·4 mm for boys).

Two-way ANOVA test observed statistically significant interaction effects of the pubertal stage and cIMT on TC (*P*= 0·035), LDL-cholesterol (*P*= 0·003) and AST (*P*= 0·033) levels (online Supplementary file). TC and LDL-cholesterol levels were significantly higher in pubertal patients with EcIMT, while AST level was significantly higher in prepubertal patients with EcIMT.

We analysed the data to determine the predictors of cIMT using linear regression. The variables age, SBP and ALT that had statistically significant correlation coefficient with cIMT at 10 % level were considered in the model. We also considered sex, BMI, presence of fatty liver and number of risk factors present in regression analysis. Since SBP was significantly correlated with age, the multi-collinearity assumption could not be met. Hence, in the final multivariate linear regression model, we included only age, sex, BMI, presence of fatty liver, number of risk factors present and ALT as independent variables. This model was not statistically significant. R^2^ = 0·032, *P*= 0·208.

No significant differences were observed in clinical and cardiometabolic parameters across different quartiles of CIMT (online Supplementary Tables 4 and 5).

## Discussion

Our study observed that nearly half of the children aged 2–15 years with overweight and obesity attending the obesity clinic of a tertiary care hospital during the study period of 2 ¼ years had EcIMT. Median ALT levels and proportion of patients with fatty liver were significantly higher in the EcIMT group compared with NcIMT group. When assessed separately, the proportion of patients with hypercholesterolemia and dyslipidemia was significantly higher in the pubertal patients with EcIMT compared with their counterparts with NcIMT and the proportion of patients with fatty liver and ≥ 3 risk factors were significantly higher in the prepubertal patients with EcIMT compared with those with NcIMT. There were statistically significant interaction effects of pubertal stage and cIMT on TC, LDL-cholesterol and AST levels.

Only one other study from South Asia compared children with overweight and obesity based on cIMT like ours. This study from Chennai, India, where 0·45 mm was taken as the cut-off for high cIMT observed EcIMT in more than 3/5th of the study participants aged 10–18 years with overweight and obesity^(6)^. EcIMT places these children at increased risk of atherosclerotic CVD in adulthood. Increased cIMT can compromise oxygen and nutrition diffusion to tissues through endothelial layers. This in turn gives rise to proliferation of small blood vessels supplying the large arterial walls which further increases the cIMT thickening leading to the initiation of endothelial dysfunction^(26)^.

Present study observed higher ALT levels and percentage of patients with fatty liver in the EcIMT group compared with NcIMT. Various studies observed higher cIMT among youth with obesity who had metabolic dysfunction-associated fatty liver disease compared with those without^(27–37)^. Prior studies in adults suggest that metabolic dysfunction-associated fatty liver disease is an independent risk factor for CVD. Proposed pathophysiological mechanism for the metabolic dysfunction-associated fatty liver disease promoted atherogenesis includes hepatic release of inflammatory cytokines, deranged lipoprotein metabolism, IR, decrease in adiponectin and increase in pro-coagulation factors^(29)^. Hence, it is not surprising that EcIMT group had higher median ALT levels and higher proportion of patients with fatty liver compared with NcIMT in our study.

Proportion of patients with ≥ 3 risk factors was significantly higher in the EcIMT group compared with NcIMT group. This was similar to the observation made by a multinational study involving 2427 children aged 6–17 years from population-based studies of Brazil, China and Italy. This study reported that the presence of one, two or at least three cardiovascular risk factors such as central obesity, elevated blood pressure, TAG and FBG and reduced HDL-cholesterol was associated with gradually increasing odds of high cIMT as compared with none. The researchers observed that the clustering of risk factors that were used to define MS predicted high cIMT more strongly than MS among children and adolescents^(38)^.

Proportion of patients with EcIMT was similar among prepubertal and pubertal children in the present study. Chennai study from India on children aged 10–18 years observed no differences in the mean cIMT according to pubertal status^(6)^. Among the pubertal patients, the proportion of patients with hypercholesterolemia, elevated LDL-cholesterol, low HDL-cholesterol and dyslipidemia was significantly higher in the EcIMT group compared with NcIMT group in our study. While the prevalence of fatty liver was higher in those with EcIMT compared with NcIMT group among the prepubertal patients, no such difference was noted between the EcIMT and NcIMT groups among the pubertal patients. Statistically significant interaction effects of cIMT and pubertal stage were noted on TC, LDL-cholesterol and AST levels: TC and LDL-cholesterol levels were significantly higher in pubertal patients with EcIMT and AST level was significantly higher in prepubertal patients with EcIMT. On our meticulous search, we did not come across other studies that analysed cIMT among prepubertal patients and pubertal patients separately. More studies exploring cardiometabolic abnormalities in children with obesity, especially cIMT and the impact of pubertal stage, are warranted.

Current study did not find any difference between the EcIMT and NcIMT groups regarding blood pressure, lipid levels, hypertension, dyslipidemia, dysglycemia, hyperinsulinemia, HOMA-IR and MS, similar to the observations made by the Chennai study on fifty children aged 10–18 years with overweight and obesity^(6)^. In contrast to our findings, a study from Germany on eighty-one children aged 6–16 years with overweight and obesity observed higher weight, BMI, BMI-standard deviation score, SBP, DBP and uric acid levels in children with cIMT ≥ 0·45 mm compared with those with cIMT < 0·45 mm^(39)^. The conflicting reports could be due to the differences in sample size and characteristics, cut-offs for cIMT used, and racial and/or ethnic characteristics between the studies.

Present study observed no statistically significant correlations between cIMT and BMI, lipid levels, liver enzymes, uric acid, glucose, HbA1c, fasting insulin and HOMA-IR in contrast to several other studies majority of which had included children with both normal and abnormal BMI^(3,7,8,39–55)^. While our findings were similar to that reported by few other studies^(48,53,56)^, studies from Egypt and Mexico on children and adolescents with obesity reported no significant correlations between cIMT and LDL-cholesterol and HDL-cholesterol and glucose and cholesterol levels, respectively^(48,53)^. Another study from Turkey on children with obesity and age and sex-matched controls reported no correlation between cIMT and lipid levels^(56)^. A systematic review on cIMT and adiposity measures such as BMI, weight status, body fat percentage and WC in children and adolescents reported that adiposity did not seem to be associated with cIMT in preadolescents (mean age < 12 years) based on studies mostly from Western Europe and USA. Out of nineteen studies on adolescents (mean age ≥ 12 years of age), thirteen observed positive associations between cIMT and adiposity measures^(57)^. The different conclusions may be explained by the non-homogenous populations studied.

Mean cIMT of the study population was 0·41 (sd 0·13) mm similar to that was reported by studies from Columbia and Egypt^(43,45)^. Compared with our observation, higher cIMT was reported by several studies on children with overweight and obesity^(2,3,6–8,26,39–42,44,46,47,49–51,55,58–70)^, while lower cIMT was reported by another study from Pune, India, on 139 children aged 6–17 years with obesity and overweight^(9)^. The differences in study population including age and racial and/or ethnic characteristics, exposure duration to obesity, sample size and the variety of ultrasound methods used for cIMT measurements may explain the difference noted between the various studies. The difference may also be due to the measurement of cIMT in various parts of the common carotid artery in these studies.

Prior studies reported reduction in cIMT with weight loss and exercise, suggesting the reversibility of early atherosclerosis and hence improvement in cardiovascular risk^(59,71)^. Wunsch *et al.* reported decrease in cIMT following considerable weight loss through 1 year of outpatient obesity intervention programme in fifty-six prepubertal children with obesity^(59)^. A meta-analysis of randomised controlled trials on effects of exercise on cIMT in children with obesity observed significant reduction in cIMT with exercise^(71)^. Pollock *et al.* observed that cardiovascular risk, as denoted by altered cIMT, can be dynamic moving between high and low cardiovascular risks over the life course of an individual. Children who betted their relative cardiovascular risk over the life course gained good midlife cardiovascular health despite having a poor metabolic health during adulthood, while those who continued to have a high cardiovascular risk throughout the life course had the worst cardiovascular risk outcome as adults^(72)^. The findings of these studies underscore the importance of efforts to change modifiable cardiovascular risk factors early in life.

Our study has several limitations, including lack of a control group, its cross-sectional study design, which does not allow causal or temporal inferences and being a single institution study. Accuracy of cIMT measurement in young children may be limited by the relatively short neck compared with the length of the US transducer and the poor compliance. Also, cut-off value for normal cIMT *v*. increased cIMT in children below 6 years was taken as the 75th percentile cIMT value for 6 years as there were no normative data available for children younger than 6 years of age^(73)^. However, the projected cIMT level of children below age 6 years based on the linear regression model developed was 0·46 mm at age 6 years which was higher than the 75th percentile cIMT value for 6 years (0·36 mm for girls and 0·4 mm for boys). There have been very few studies from South Asia assessing cIMT in children with obesity and overweight^(6–9)^, and ours is the first study from Asia assessing cIMT in children below 5 years of age with obesity and overweight.

### Conclusions

Our finding of EcIMT in nearly half of the study participants including young children is very concerning as these children are at increased risk of atherosclerotic CVD in adulthood. Public health efforts are needed in early childhood to mitigate overweight and obesity to avoid associated cardiometabolic risks that are already emerging in childhood. Early interventions are important when trajectories are likely to be more malleable and adverse cardiometabolic phenotypes and subclinical atherosclerosis are reversible.

## Supporting information

Sasidharan Pillai et al. supplementary material Sasidharan Pillai et al. supplementary material

## Data Availability

Some or all datasets generated during and/or analysed during the current study are not publicly available but are available from the corresponding author on reasonable request.

## Supplementary material

For supplementary material/s referred to in this article, please visit https://doi.org/10.1017/S0007114525000091
