# Supplementary material for: Carotid intima medial thickness and its association with cardiometabolic risk factors in children with overweight and obesity: a hospital-based cross-sectional study
Source: Br J Nutr. 2025 Jan 27;133(4):456–64. doi: 10.1017/S0007114525000091 (PMC12011544; doi:10.1017/S0007114525000091)
Supplement: Sasidharan Pillai et al. supplementary material [file S0007114525000091sup001.docx]

**Supplemental Table 1. Cardiometabolic abnormalities in prepubertal and pubertal patients with normal vs. elevated cIMT**

| Clinical and metabolic parameters | Prepubertal (n=129) | | | Pubertal (n=94) | | |
| --- | --- | --- | --- | --- | --- | --- |
|  | NcIMT (n=71) | EcIMT (n=58) | p value | NcIMT (n=50) | EcIMT  (n=44) | p value |
| Central Adiposity (n (%)) | 69 (97.2) | 56 (96.6) | 0.837 | 48 (96.0) | 40 (90.9) | 0.314 |
| Hypertension (n(%)) | 10 (14.1) | 8 (13.8) | 0.962 | 42 (84.0) | 35 (79.5) | 0.576 |
| Elevated SBP (n(%)) | 3 (4.2) | 3 (5.2) | 1.0 | 4 (8.0) | 7 (15.9) | 0.234 |
| Elevated DBP (n(%)) | 9 (12.7) | 7 (12.1) | 0.917 | 5 (10.0) | 6 (13.6) | 0.584 |
| Elevated ALT (n(%)) | 33 (46.5) | 35 (60.3) | 0.117 | 23 (46.)) | 20 (45.5) | 0.958 |
| Hyperuricemia (n(%)) | 1 (1.4) | 1(1.7) | 0.885 | 2 (4.0) | 2 (4.5) | 0.896 |
| IFG (n(%)) | 6 (8.5) | 6 (10.3) | 0.713 | 7 (14.0) | 2 (4.5) | 0.120 |
| Elevated HbA1c (n(%)) | 20 (28.2) | 20 (34.5) | 0.441 | 13 (26.0) | 11 (25.0) | 0.912 |
| Elevated HOMA-IR (n (%) | 36 (50.7) | 27 (46.6) | 0.639 | 39 (78.0) | 29 (65.9) | 0.191 |
| Hyperinsulinism (n (%) | 16 (22.5) | 20 (34.5) | 0.132 | 9 (18.0) | 9 (20.5) | 0.763 |
| Hypercholesterolemia (n (%)) | 19 (26.8) | 14 (24.1) | 0.734 | 8 (16.0) | 16 (36.4) | 0.024 (0.396) |
| Hypertriglyceridemia (n (%)) | 31(43.7) | 27 (46.6) | 0.743 | 19 (38.0) | 23 (52.3) | 0.165 |
| Low HDL-C (n (%)) | 24 (33.8) | 21 (36.2) | 0.776 | 10 (20.0) | 18 (40.9) | 0.027 (0.133) |
| Elevated LDL-C (n (%)) | 21 (29.6) | 11 (9.0) | 0.165 | 8 (16.0) | 17 (38.6) | 0.013 (0.434) |
| Dyslipidemia (n (%)) | 50 (70.4) | 35 (60.3) | 0.230 | 29 (58.0) | 37 (84.1) | 0.006 (0.345) |
| Fatty liver (n (%)) | 32 (45.1) | 37 (63.8) | 0.034 | 27 (54.0) | 28 (63.6) | 0.344 |

ALT- alanine transaminase; DBP-diastolic blood pressure; EcIMT-elevated carotid intima media thickness; IFG-impaired fasting glucose; HbA1c- hemoglobin A1c; HDL-C – high density lipoprotein cholesterol; HOMA-IR- homeostatic model assessment of insulin resistance; LDL-C- low density lipoprotein cholesterol; NcIMT-normal carotid intima media thickness; SBP- systolic blood pressure

Adjusted p values are given in the bracket.

**Supplemental Table 2. Correlation between cIMT and clinical and cardiometabolic parameters in prepubertal and pubertal patients**

| Clinical & cardiometabolic parameter | Prepubertal patients (n=129) | | Pubertal patients (n=94) | |
| --- | --- | --- | --- | --- |
|  | Correlation coefficient (r) | p value | Correlation coefficient (r) | p value |
| Age^*^ | 0.11 | 0.23 | 0.04 | 0.69 |
| BMI | 0.03 | 0.71 | 0.02 | 0.84 |
| Birth weight | -0.13 | 0.88 | 0.09 | 0.38 |
| WC | 0.05 | 0.60 | -0.04 | 0.73 |
| SBP^*^ | 0.13 | 0.13 | 0.04 | 0.68 |
| DBP^*^ | 0.02 | 0.82 | -0.03 | 0.76 |
| AST^*^ | 0.03 | 0.78 | -0.13 | 0.22 |
| ALT^*^ | 0.12 | 0.18 | 0.12 | 0.89 |
| Uric acid^*^ | 0.05 | 0.57 | -0.13 | 0.19 |
| FBG | 0.01 | 0.93 | -0.12 | 0.25 |
| HOMA-IR^*^ | 0.02 | 0.86 | -0.12 | 0.27 |
| Fasting insulin^*^ | 0.04 | 0.63 | -0.09 | 0.40 |
| HbA1c^*^ | 0.04 | 0.68 | 0.05 | 0.65 |
| TC | -0.07 | 0.44 | 0.13 | 0.23 |
| Triglycerides ^*^ | 0.01 | 0.31 | 0.08 | 0.44 |
| LDL-C | -0.14 | 0.13 | 0.17 | 0.11 |
| VLDL-C^*^ | 0.10 | 0.26 | 0.04 | 0.67 |
| HDL-C | 0.09 | 0.32 | 0.14 | 0.20 |

ALT- alanine transaminase; AST-aspartate transaminase; BMI- body mass index; cIMT-carotid intima media thickness; DBP-diastolic blood pressure; FBG- fasting blood glucose; HbA1c- hemoglobin A1c; HDL-C – high density lipoprotein cholesterol; HOMA-IR- homeostatic model assessment of insulin resistance; LDL-C- low density lipoprotein cholesterol; SBP- systolic blood pressure; TC- total cholesterol; VLDL-C – very low density lipoprotein cholesterol; WC- waist circumference.

*Spearman’s correlation coefficient, others Pearson correlation coefficient

**Supplemental Table 3. Correlation between cIMT and clinical and cardiometabolic parameters in children below 6 years**

| Clinical & cardio metabolic parameters | Correlation coefficient (r) | p value |
| --- | --- | --- |
| Age | 0.454 | 0.03 (0.03) |
| BMI | 0.06 | 0.786 |
| Birth weight | -0.003 | 0.989 |
| WC | 0.125 | 0.571 |
| SBP | 0.054 | 0.808 |
| DBP | 0.102 | 0.643 |
| AST | 0.031 | 0.888 |
| ALT^*^ | 0.043 | 0.847 |
| Uric acid | 0.199 | 0.362 |
| FBG | 0.137 | 0.534 |
| HOMA-IR^*^ | -0.106 | 0.631 |
| Fasting insulin^*^ | -0.14 | 0.523 |
| HbA1c | -0.049 | 0.825 |
| TC^*^ | -0.026 | 0.905 |
| Triglycerides ^*^ | 0.227 | 0.298 |
| LDL-C^*^ | -0.151 | 0.493 |
| VLDL-C^*^ | 0.281 | 0.194 |
| HDL-C | 0.158 | 0.473 |

ALT- alanine transaminase; AST-aspartate transaminase; BMI- body mass index; cIMT-carotid intima media thickness; DBP-diastolic blood pressure; FBG- fasting blood glucose; HbA1c- hemoglobin A1c; HDL-C – high density lipoprotein cholesterol; HOMA-IR- homeostatic model assessment of insulin resistance; LDL-C- low density lipoprotein cholesterol; SBP- systolic blood pressure; TC- total cholesterol; VLDL-C – very low density lipoprotein cholesterol.

Adjusted p value is given in bracket.

*Spearman’s correlation coefficient, others Pearson correlation coefficient

**Supplemental Table 4. Clinical and demographic factors by quartiles of cIMT**

| Clinical & metabolic parameters | cIMT(in mm) (n=223) | | | | p value |
| --- | --- | --- | --- | --- | --- |
|  | ≤ 0.30 (n=70) | 0.30 -0.40 (n=54) | 0.41-0.50 (n=49) | ≥ 0.51(n=50) |  |
| Age, years (median ± IQR) | 10.1 ± 3.8 | 11.2 ± 4.1 | 10.3 ± 4.1 | 10.6 ± 3.2 | 0.11 |
| 2-5 years (n (%)) | 7 (10) | 6 (11.1) | 8 (16.3) | 2 (4) | 0.082 |
| 6-10 years(n(%)) | 42 (60) | 20 (37) | 21(42.9) | 26 (52) |  |
| 11-15 years(n(%)) | 21(30) | 28 (51.9) | 20 (40.8) | 22 (44) |  |
| Female (n(%)) | 30 (42.9) | 17 (31.5) | 22 (44.9) | 15 (30) | 0.259 |
| Male (n(%)) | 40 (57.1) | 37 (68.5) | 27 (55.1) | 35 (70) |  |
| Overweight(n(%)) | 11 (15.7) | 13(24.1) | 8(16.3) | 11(22) | 0.597 |
| Obesity(n(%)) | 59 (84.3) | 41 (75.9) | 41 (83.7) | 39(78) |  |
| Prepubertal(n(%)) | 44 (62.9) | 29 (53.7) | 33 (67.3) | 23 (46) | 0.122 |
| Pubertal(n(%)) | 25 (37.1) | 26 (46.3) | 26 (32.7) | 27 (54) |  |
| FH of hypertension (n (%)) | 47 (67.1) | 38 (70.4) | 27 (55.1) | 21 (42) | 0.305 |
| FH of dyslipidemia(n (%)) | 39 (55.7) | 30 (55.6) | 27 (55.1) | 22 (44) | 0.560 |
| FH of T2D (n(%)) | 51(72.9) | 35 (64.8) | 36 (73.5) | 38 (76) | 0.606 |
| FH of heart attack (n(%)) | 24 (34.3) | 28 (51.9) | 21 (42.9) | 14 (28) | 0.064 |
| BMI, kg/m^2^ (mean ± SD) | 23.8 ± 3.0 | 24.2 ± 3.6 | 24.1 ± 3.0 | 24.5 ± 3.6 | 0.783 |
| BW, kg (mean ± SD) | 3.0 ± 0.7 | 3.1 ± 0.6 | 2.8 ± 0.6 | 3.1 ± 0.5 | 0.227 |
| WC, cm (mean ± SD) | 83.3 ± 9.4 | 84.4 ± 9.4 | 83.7 ± 9.8 | 84.8 ± 9.3 | 0.828 |
| SBP, mm Hg (median ± IQR) | 100.0 ± 0.0 | 100.0 ± 10.0 | 100.0 ± 10.0 | 101.0 ± 17.0 | 0.335 |
| DBP, mm Hg (median ± IQR) | 62.0 ± 10.0 | 63.5 ± 10.0 | 60 ± 17.0 | 64 ± 10.0 | 0.851 |
| AST, IU/L (median ± IQR) | 26.7 ± 12.5 | 24.0 ± 9.3 | 31.0 ± 15.5 | 24.0 ± 9.2 | 0.28 |
| Uric acid, mg/dL (median ± IQR) | 3.8 ± 0.8 | 3.8 ± 1.3 | 3.6 ± 1.4 | 3.7 ± 1.0 | 0.946 |
| FBG, mg/dL (mean ± SD) | 87.6 ± 12.4 | 85.6 ± 10.3 | 84.6 ± 8.1 | 85.6 ± 8.8 | 0.463 |
| HOMA-IR (median ± IQR) | 2.8 ± 2.6 | 2.7 ± 1.7 | 2.6 ± 2.2 | 3.3 ± 3.2 | 0.602 |
| Fasting Insulin mIU/L (median ± IQR) | 13.6 ± 10.8 | 12.7 ± 8.2 | 13.1 ± 10.5 | 15.1 ± 13.7 | 0.575 |
| HbA1c, % (median ± IQR) | 5.3 ± 0.6 | 5.4 ± 0.5 | 5.4 ± 0.6 | 5.4 ± 0.7 | 0.827 |
| TC, mg/dL (mean ± SD) | 181.0 ± 3.2 | 169.9 ± 28.1 | 185.3 ± 32.8 | 175.4 ± 34.0 | 0.088 |
| TG, mg/dL (median ± IQR) | 94.5 ± 55.8 | 109.0 ± 79.5 | 121.0 ± 74.0 | 107.6 ± 86.8 | 0.191 |
| LDL-C, mg/dL (mean ± SD) | 115.0 ± 33.2 | 104.0 ± 25.1 | 120.1 ± 30.1 | 107.8 ± 27.0 | 0.291 |
| VLDL-C, mg/dL (median ± IQR) | 18.7 ± 10.2 | 21.2 ± 14.3 | 25.0 ± 14.0 | 20.1 ± 15.9 | 0.359 |
| HDL-C, mg/dL (mean ± SD) | 43.5 ± 7.9 | 41.9 ± 7.6 | 41.0 ± 8.0 | 43.8 ± 9.6 | 0.249 |

AST-aspartate transaminase; BMI- body mass index; cIMT-carotid intima media thickness; DBP-diastolic blood pressure; FH- family history; FBG –fasting blood glucose; HbA1c- hemoglobin A1c; HDL-C – high density lipoprotein cholesterol; HOMA-IR- homeostatic model assessment of insulin resistance; IFG- impaired fasting glucose; LDL-C- low density lipoprotein cholesterol; SBP- systolic blood pressure; TC- total cholesterol; VLDL-C – very low density lipoprotein cholesterol; WC- waist circumference

**Supplemental Table 5. Cardiometabolic abnormalities by quartiles of cIMT**

| Clinical and metabolic parameters | cIMT(in mm) | | | | p value |
| --- | --- | --- | --- | --- | --- |
|  | ≤ 0.30 (n=70) | 0.30 -0.40 (n=54) | 0.41-0.50 (n=49) | ≥ 0.51(n=50) |  |
| Central Adiposity (n (%)) | 68 (97.1) | 51(94.4) | 48 (98) | 46 (92.0) | 0.440 |
| Hypertension (n(%)) | 8 (11.4) | 10 (18.5) | 12 (24.5) | 5 (10.0) | 0.146 |
| Elevated SBP* | 4 (5.7) | 3 (5.6) | 8 (16.3) | 2 (4.0) | 0.259 |
| Elevated DBP | 6 (8.6) | 8 (14.8) | 9 (18.4) | 4 (8.0) | 0.285 |
| Elevated ALT (n(%)) | 34 (48.6) | 24 (44.4) | 29 (59.2) | 24 (48.0) | 0.484 |
| Hyperuricemia (n(%)) | 2 (2.9) | 1(1.9) | 1(2) | 2 (4.0) | 0.906 |
| IFG (n(%)) | 10 (14.3) | 4 (7.4) | 2 (4.1) | 5 (10.0) | 0.277 |
| Elevated HbA1c (n(%)) | 18 (25.7) | 17 (31.5) | 14 (28.6) | 15 (30.0) | 0.908 |
| Elevated HOMA-IR (n (%) | 45 (64.3) | 31 (57.4) | 28 (57.1) | 27 (54.0) | 0.695 |
| Hyperinsulinism (n (%) | 18 (25.7) | 7 (13) | 15 (30.6) | 14 (28.0) | 0.151 |
| Hypercholesterolemia(n (%)) | 17 (24.3) | 11 (20.4) | 20 (40.8) | 9 (18.0) | 0.40 |
| Hypertriglyceridemia (n (%)) | 23 (32.9) | 29 (53.7) | 26 (53.1) | 22 (44.0) | 0.068 |
| Low HDL-C*(n (%)) | 18 (25.7) | 17 (31.5) | 22 (44.9) | 16 (32.0) | 0.179 |
| Elevated LDL-C* (n (%)) | 19 (27.1) | 10 (18.5) | 20 (40.8) | 8 (16.0) | 0.190 |
| Dyslipidemia (n (%)) | 45 (64.3) | 36 (66.7) | 38 (77.6) | 32 (64.0) | 0.409 |
| Fatty liver (n (%)) | 34 (48.6) | 26 (48.1) | 34 (69.4) | 30 (60.0) | 0.079 |

ALT- alanine transaminase; DBP-diastolic blood pressure; HbA1c- hemoglobin A1c; HDL-C – high density lipoprotein cholesterol; HOMA-IR- homeostatic model assessment of insulin resistance; IFG- impaired fasting glucose; LDL-C- low density lipoprotein cholesterol; SBP- systolic blood pressure.

*data pooling done since expected cell frequency was < 5
